# Adapting Patient and Public Involvement processes in response to the Covid‐19 pandemic

**DOI:** 10.1111/hex.13771

**Published:** 2023-05-01

**Authors:** Claire Snowdon, Elizabeth Silver, Paul Charlton, Brian Devlin, Emma Greenwood, Andrew Hutchings, Susan Moug, Ravinder Vohra, Richard Grieve

**Affiliations:** ^1^ Department of Medical Statistics, London School of Hygiene and Tropical Medicine University of London London UK; ^2^ Women and Children First London UK; ^3^ ESORT Studies London UK; ^4^ Patient and Public Involvement (PPI) London UK; ^5^ Department of Health Services Research and Policy London School of Hygiene and Tropical Medicine London UK; ^6^ Department of Surgery Royal Alexandra Hospital Paisley Renfrewshire UK; ^7^ Trent Oesophago‐Gastric Unit, Nottingham University Hospitals NHS Trust City Hospital Campus Nottingham UK; ^8^ Nottingham Digestive Diseases Centre, National Institute for Health Research (NIHR) Nottingham Biomedical Research Centre, Nottingham University Hospitals NHS Trust Queen's Medical Centre Nottingham UK

**Keywords:** COVID‐19, e‐PPI, emergency surgery, online consultation methods, patient and public involvement, remote methods

## Abstract

**Background:**

The COVID‐19 pandemic brought rapid and major changes to research, and those wishing to carry out Patient and Public Involvement (PPI) activities faced challenges, such as restrictions on movement and contact, illness, bereavement and risks to potential participants. Some researchers moved PPI to online settings during this time but remote consultations raise, as well as address, a number of challenges. It is important to learn from PPI undertaken in this period as face‐to‐face consultation may no longer be the dominant method for PPI.

**Methods:**

UK stay‐at‐home measures announced in March 2020 necessitated immediate revisions to the intended face‐to‐face methods of PPI consultation for the ESORT Study, which evaluated emergency surgery for patients with common acute conditions. PPI plans and methods were modified to all components being online. We describe and reflect on: initial plans and adaptation; recruitment; training and preparation; implementation, contextualisation and interpretation. Through first‐hand accounts we show how the PPI processes were developed, experienced and viewed by different partners in the process.

**Discussion and Conclusions:**

While concerns have been expressed about the possible limiting effects of forgoing face‐to‐face contact with PPI partners, we found important benefits from the altered dynamic of the online PPI environment. There were increased opportunities for participation which might encourage the involvement of a broader demographic, and unexpected benefits in that the online platform seemed to have a ‘democratising’ effect on the meetings, to the benefit of the PPI processes and outcomes. Other studies may however find that their particular research context raises particular challenges for the use of online methods, especially in relation to representation and inclusion, as new barriers to participation may be raised. It is important that methodological challenges are addressed, and researchers provide detailed examples of novel methods for discussion and empirical study.

**Patient and Public Contribution:**

We report a process which involved people with lived experience of emergency conditions and members of the public. A patient member was involved in the design and implementation, and two patients with lived experience contributed to the manuscript.

## 1 INTRODUCTION

In 2019, the National Institute of Health Research (NIHR) published UK Standards for Public Involvement in Research,^1^ to support high‐quality Patient and Public Involvement (PPI), and encourage growth and methodological development. In 2020, the COVID‐19 pandemic brought rapid and major changes to research, and in England, PPI was said to have been ‘sidelined’.^2^ The Health Research Authority (HRA) reported that only 20% of studies submitted in March–April 2020 incorporated PPI compared with 80% of studies prepandemic.^3^ Reviews of HRA applications suggested an ‘absence of a clear shared understanding of what is feasible and beneficial in terms of public involvement during a public health crisis’; researchers were ‘postponing or even cancelling PPI activities’ especially where there were known vulnerabilities in potential PPI partners.^4^ This has potential implications for research quality, fairness and representation.^5^ PPI empowers people by representing their views within research and is considered a form of social justice.^6^ Following changes within society and in applied research since the COVID‐19 pandemic, face‐to‐face consultation may no longer be considered the most appropriate approach to PPI.

### 1.1 PPI in a changing context

There is a small and varied evidence base showing changes to PPI processes made during the COVID‐19 pandemic. Some teams created new partnerships. Jamal and colleagues report how a rapidly assembled PPI panel changed the design of a COVID‐19 trial for patients with cardiovascular disease, high blood pressure and diabetes.^7^ After a one hour online meeting with patients living with these co‐morbidities, the team made alterations to address concerns over the planned withdrawal of prescribed medication. Other teams worked with existing relationships. Adeyemi and colleagues^8^ were conducting PPI with marginalised groups with vulnerabilities; they developed strategies should partners become distressed or unwell during online meetings, and, for familiarity, mirrored methods used in previous face‐to‐face meetings. Leese and colleagues emphasised the importance of sustaining ‘social connections and trust that had been built over time’.^6^

Online group dynamics can be complex. Lampa and colleagues^5^ explored a shift online by observing PPI meetings with people with experience of seeking refuge, and those facing economic hardship. They saw the online meetings as more structured and facilitators more directive than in their previous observations of face‐to‐face meetings. With fewer spontaneous interactions and nonverbal cues, increased linguistic barriers and difficulties in claiming space to speak, they conclude that online PPI is possible but requires adaptations to ‘solve practical issues’. For PPI with groups of adults with visual impairment Adeyemi and colleagues^8^ invested time developing context‐appropriate online methods, including very small groups, planned turn‐taking for speaking, avoiding visual cues, and using spoken summaries. Rasburn and colleagues^9^ found that PPI participants reported a ‘sense of comfort’ and ‘a reduced feeling that involvement is daunting’ which they felt related to control over the online environment, for example, volume control, full‐screen views of speakers.

This paper aims to describe how PPI processes were modified during the pandemic, within the context of an evaluation of emergency surgery for common acute conditions. We focus on initial PPI plans and adaptation; recruitment; training and preparation; implementation, contextualisation and interpretation. Through first‐hand accounts we show how the processes were developed, experienced and viewed by different partners. We then reflect on central themes that emerged in the context of related literature.

## 2 METHODS

### 2.1 The Emergency Surgery OR noT (ESORT) Studies—Initial PPI plans and adaption

The ESORT Studies consider the cost‐effectiveness of emergency surgery for patients admitted to NHS hospitals with common acute conditions (appendicitis, gallstone disease, diverticular disease, small bowel obstruction, abdominal hernia).^10^, ^11^ The studies use Hospital Episode Statistics (HES) data to compare outcomes and costs of emergency surgery and other approaches to care (medical management, nonsurgical procedures or planned surgery). The initial NIHR‐funded ESORT Study ran from October 2019 to October 2021, with further funding awarded by The Health Foundation for ESORT‐C19^12^ to assess the impact of the COVID‐19 pandemic on clinical management and outcomes of patients admitted to hospitals with these conditions.

PPI plans were developed, but not implemented, due to the stay‐at‐home measures announced in March 2020. Plans included two face‐to‐face meetings with a panel with lived experience of emergency surgery for common acute conditions, carers and members of the public. The first meeting (A) was intended to reflect on study outcome measures, and the second meeting (B) was the communication of research findings. They were planned as full‐day events in London, comprising training with presentations on research aims and methods (A), and research findings (B), activities and panel discussions.

The revisions to the plans were:

1. meetings were moved online, shortened to two 2‐h sessions with smaller groups;
2. the target number of panellists was increased from 10 to 12–16 people, enabled by lower costs of online meetings;
3. a choice of meeting times was offered (afternoon or evening);
4. asynchronous self‐managed training was developed.

**Figure 1:**
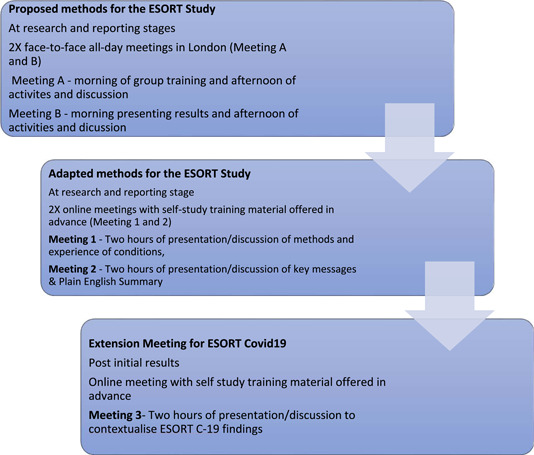
Planned and adapted methods for The ESORT Study and ESORT‐C19. ESORT, Emergency Surgery OR noT.

These adaptations were carried over to ESORT‐C19. To differentiate the planned (A and B) and adapted meetings, we refer to the latter as meetings 1, 2 and 3 (Figure 1).

### 2.2 Recruitment to the panels

Panellists were recruited in May 2020 via multiple routes. Moving the meetings online meant that ESORT Study clinicians were able to share recruitment invitations with their local patient groups regardless of proximity to London. Members of the public were recruited via the NIHR Applied Research Collaboration network, and contacts of the study's Patient Members.

Fourteen panellists joined the initial meetings held in July 2020. There were two further meetings in September 2021 and two in May 2022. One member left the panel. Another who was unable to make earlier dates joined the final meeting.

### 2.3 Recruitment of PPI panellists into the authorship group

Two panellists were later invited to be co‐authors of this paper. B. D. had been clear that the online meetings had given him the opportunity to participate. E. G. had given a compelling account of emergency care during the pandemic in meeting 3. While we acknowledge that other experiences will have existed within the panel, these were important positions to explore. They, and three ESORT team members, a clinician (S. M.), the Principal Investigator (R. G.) and the ESORT Study Patient Member (P. C.), contributed text relating to their involvement.

The five unedited contributions are reported in full throughout this paper and speak to key issues relating to design and implementation, and raise issues for further reflection in the discussion. An example is that of widening recruitment and representation as raised by B. D.

I feel that a lot of patients who live in rural communities probably don't get to participate in these sort of exercises because of where they live. I live in a rural area some considerable distance from London, so realistically, even without a pandemic, I would not have travelled to attend the sessions. I get the impression that these studies stray into an urban mindset where there are plenty of opportunities for people to access healthcare settings. So being able to champion rurality is something that I do, and I think it is an important take. (B. D.)

### 2.4 Training and preparation

Before the first meetings, panellists were sent bespoke training materials via an online link, and a publicly available document explaining Zoom and online meeting etiquette. The team felt that clear and engaging information was essential to support the discussion. The training materials were created using Microsoft Sway which can create a visually appealing and information‐rich online document with text, images, videos and embedded links to resources such as websites or publications.

The content was co‐produced by the PPI lead, the Patient Member who produced a video, the clinicians and academics. The text covered key topics and was written in plain English and to promote familiarity, images of the ESORT team member who would present and lead discussions on each topic were included. Powerpoint was used to create meeting slides.

Sway and Powerpoint include accessibility checkers which prompt users to describe images with alternative text and embed hyperlinks into the meaningful text to support anyone using a screen reader and to limit repetition. Panellists were advised that accessible versions could be viewed, exported and printed (support was offered), or the ESORT team could post copies. Before meeting 1, a panellist indicated that support would be helpful. They wished to work through the materials independently but met online with C. S. to go over the slides with their screen reader to process information and tasks and consider responses in advance. Later, it became clear that another panellist might have benefited from similar support. Support was offered to both panellists for meeting 2 but was not needed.

Training materials were tailored to each meeting.

Meeting 1 materials (July 2020)^13^ covered

1. the role of PPI;
2. the Patient Member video;
3. the research question—*is it better for patients with common acute conditions, such as appendicitis or gallstones, to have emergency surgery, or other types of care?*
4. key terms and concepts, for example, emergency surgery, HES data, outcome measures, quality of life;
5. explanations of three common conditions that lead to emergency admission. Appendicitis, gallstone disease and diverticular disease were described through written descriptions, publicly available videos, and illustrated narrative accounts referred to as ‘patient stories’;
6. the plan for the PPI meeting, and details of the tasks.

Meeting 2 materials (September 2021)^14^:

1. revisited ESORT Study methods, and described the results;
2. hyperlinked to meeting 1 materials to refresh knowledge of the conditions and patient stories;
3. presented a draft Plain English Summary of results for revision in the meeting;
4. presented questions to be discussed in the meeting.

Meeting 3 materials (May 2022)^15^:

1. described the UK COVID‐19 pandemic timeline;
2. revisited ESORT Study and ESORT C‐19 methods, and described the results;
3. hyperlinked to meeting 1 materials to refresh knowledge of the conditions and patient stories;
4. presented questions to be discussed in the meeting.

The materials were intended to convey information and deepen learning, to prepare the panel for meeting tasks and discussion. They could be worked through at an individual pace. Embedded extras could be explored or skipped. Earlier materials could be revisited. They met different learning styles, as per the VARK model^16^: **V**isual learners prefer to learn by seeing and observing, and charts, graphs and figures were included; **A**uditory learners learn by listening and videos were used to consolidate and extend other material; text‐supported **R**eading learners and for **K**inesthetic learners who learn best with a task, information cards were clicked to reveal hidden content. A house style was maintained in the materials and slides for continuity and to foster familiarity over time.

B. D. saw the training materials as a key part of his involvement.

I am a frequent, and generally very enthusiastic, participant in PPI sessions. I think, having received excellent surgical care throughout my life within the NHS, I have a responsibility to do all I can to participate in anything that needs patient input. But, and there is a caveat to that responsibility, the input – my time, effort (frequently when unwell – people must remember that by dint of being a patient one is occasionally sick) and ability to respond does need to feel valued? I don't know if that feeling can be measured but it is very important. And, boy, do you notice when it isn't there in full

I fit two of the criteria for participating ‐ parastomal hernia and gallbladder removal – so I was prepared to give it my all. I am extremely glad that I did. It is one of the PPI sessions that I have had the best experience with. The staff have been so positive and kind. And extremely responsive

I've participated in PPI sessions where the preparatory literature was non‐existent, incomprehensible or poorly written. As an author good writing is important to me. The writing for this study, alongside the pictures, and infographics was outstanding. It was easily the best that I have read. It was a joy to consume– and I took my time with it rather than cram it half an hour before the Zoom calls. (B. D.)

### 2.5 Implementation

The meetings were carried out on Zoom, which offers functionality well‐suited to our needs. Participant images and names are displayed if they choose. Reaction tools allow hand‐raising to request to speak or to support or disagree with a point. Images move to the top of the display when someone speaks or reacts allowing chairs to be responsive.

The meetings opened early for virtual coffee, paralleling opportunities for an informal chat before face‐to‐face meetings. The screen showed an image of a coffee cup. Meetings included introductions, presentations, questions and tasks for discussion. A screen break mid‐way closed opportunities for further informal contact but seemed important in a session exceeding two hours. While there was a facilitator, each section was handled by different ESORT team members with the expertise to present, answer and reflect on questions, so there was not one dominant voice.

Meeting tasks were:

1. discussion of ESORT outcome measures, and consideration of extending ESORT to consider the impact of COVID‐19;
2. discussion and refinement of the Plain English Summary;
3. contextualisation of HES data from the first wave of the pandemic.

The first meetings ran a tight schedule. Later meetings had more space for discussion and likely benefited from increasing familiarity with the research, the team, meeting methods and the panel. An ESORT clinician reflects below on the possibility that engagement with the materials affected the feel of the meeting.

The prep work online was novel and had clearly been read by the participants. I wonder if this meant fewer questions on clinical context or terminology or practice than usual. In addition to improving understanding the conversation with everyone started at the beginning ‐ did this prep get over awkwardness, or that feeling that the clinicians have just given me too much info and I'm not sure if I'm asking a wrong or silly question?

This work is really hard to understand and when questions were asked of us it felt like a conversation again. Some PPI work I have done moves quickly into patient experience with some difficult questions about their care ‐ this can be confrontational for the surgeon. Didn't feel like this at all

The chair or moderator used first names and with us not doing a presentation first, the usual hierarchy is not there. Also felt easy to ask the patients questions back. (S. M.)

After each meeting, panellists were thanked with online shopping vouchers (following INVOLVE guidelines^17^). B. D. commented:

I want to make the point that patients are frequently the only unpaid participants in the ‘room’. The ESORT study recognised our input's value through two generous gift tokens. Any reasonable sort of payment does help with that unmeasured feeling I spoke about. PPI can often feel like an afterthought. The ESORT studies were exceptional at making us feel part of it all. I'm so glad that I was a part of it. (B. D.)

### 2.6 Contextualisation and interpretation

Meeting 3 aimed to contextualise initial ESORT‐C19 findings, to drive forward interpretation and reflection within the study team. The study identified a large reduction in emergency admissions and worse outcomes for acute conditions during the first wave of the pandemic, versus corresponding 2019 admissions. It was therefore important to hear from patients who lived with the conditions during the pandemic, and from those providing surgical care at that time. Shared experiences of the pandemic triggered an extended group discussion.

The online meeting did not inhibit sharing. In meeting 3, E. G. gave a powerful account of decision‐making for a shielding patient under pandemic conditions, which provided important context to the ESORT‐C19 Study. For this paper, E. G. contributed text which gives a sense of the rich informative material that had been available in online PPI.

I got involved in ESORT to improve future care by sharing my experiences as a shielding patient during COVID. The online meetings allowed me to contribute because my family life, work, and, particularly, illness would have made it too unpredictable to allow me to travel to London. I think face‐to‐face meetings would have been more stressful. We were given clear information and guiding questions to think about in advance. The meetings were a welcoming environment, where everyone was given the chance to contribute relevant information

I have had severe inflammatory bowel disease for eight years requiring multiple admissions for medical and surgical treatment. I became unwell during the first lockdown and required urgent surgery. Covid had a big impact on the care that I received. The decision on when to get help was hard; the overwhelming message from the media and clinicians was to avoid hospitals, GPs and A&E. But it was less clear how you should get help when it was needed. Regular monitoring of chronic diseases is much harder when face‐to‐face clinics are discouraged and you are left to make decisions alone about whether the deterioration in your health outweighs the risk of getting help. The lockdown also made it harder to get help with childcare to be able to attend healthcare facilities

During the first wave staff were very stressed. There were fewer junior doctors on the ward and an atmosphere of fear. Because of the risk of severe Covid, and the lack of critical care beds, the available surgical options were more conservative and more limited. This meant that I had minimal surgery to control the acute emergency, and would have to wait while unwell and in pain, until the full operation could be done safely with critical care back‐up

When I was first admitted I was sent home again to avoid Covid until my consultant could get a theatre slot two days later. I was very unwell at home during this period, whereas normally I would have stayed in, receiving treatment. It was stressful being anaesthetised in theatre. It was cold and scary lying on the operating table awake with all the instrument trolleys around you and unable to communicate well with the staff in full PPE. Post‐op I was discharged earlier than normal, a joint decision to reduce exposure, which again meant being more unwell at home than usual. However, I did have access to surgical hot clinic which provided outstanding aftercare and a follow up contact service. I required a further operation a few months later

There has been an unknown amount of suffering at home with reduced routine care of chronic diseases, delays accessing treatment and earlier discharge. This for me led to multiple re‐admissions, more time off work for me and my partner, more stress for the family, delayed recovery, more pain, worse disease management and worse outcome from surgery. At the point when I accessed emergency care, I was sicker, less optimised for surgery and suffered more complications. I had longer recovery times and more readmissions. I had further surgeries after the lock downs as was unable to have the surgery I needed electively during the lockdown periods due to increased risk, lack of critical care beds and reduced operating. (E. G.)

The meetings elicited first‐hand accounts from patients and clinicians and fostered discussion and reflection. For instance, one panellist explained that they had been given postoperative support for recovery prepandemic, but during the pandemic, more time was spent in bed due to staffing constraints. This comparison was available to someone with lived experience of surgery before and during the pandemic but was new to the academics. Panellists asked questions about the ESORT findings. These helped to develop a better interpretation of the findings, as the PI explains below.

The meetings allowed the research team and panellists to raise questions of one another about the initial study findings. Sharing experiences triggered some useful discussions. In particular, the panellists highlighted the need for the research team to make it clear that the excess deaths were unlikely to be attributed to individual patients who had COVID‐19, and more likely due to the disruption to the health care system. Panellists also shared their experiences of attending hospital during the first wave of COVID‐19 and supported the suggestion that fear of infection could have led to fewer patients presenting to hospital, and so those who did had more advanced disease. These thoughts helped us to redraft the discussion of the findings to emphasise that patients and the public needed information about the risks of not attending hospital at times of crisis. These discussions between panellists, clinical colleagues and those undertaking the analyses can collectively identify key messages, especially for studies like ESORT that use routine datasets. Otherwise researchers may be somewhat ‘distant’ from the patients who will benefit from the evidence generated. (R. G.)

### 2.7 Co‐production

As a final point of reflection, the Patient Member who was involved in the studies from the prefunding stage focussed on ‘co‐production’ as a continuous approach in the design, revision and implementation of the research.^18^ The shift online was incorporated into this approach with shared development of the training materials as described above, detailed session planning, and the hosting of some sessions by the Patient Member.

Co‐production happens well when everyone involved in the activity works together and recognises each other as being equally valuable. The ESORT research study is about understanding surgical decision‐making and the treatment outcomes following those decisions. Experiencing those treatment outcomes are the patients and their family carers. This means that, once decided upon approaching their work with a co‐production intention, the ESORT lead researchers necessarily set about having a range of patients and family carers also active in the study design, data analysis and dissemination of the findings. The co‐production challenge to ESORT was that it is about routinely collected patient data. How do you bring patients and family carers into an abstract data analysis study? Would there be an energy and a desire from patients to grapple with data? Co‐production in fact values different kinds of knowledge and evidence. Collecting data tells us about patterns across the decision and treatment surgical care system that patient storytelling can't reveal. But patient storytelling reveals personal experiences which collecting data can't tell us about. The ESORT studies ensured everyone brought value both from their lived experience as patients and from their learned knowledge as clinicians and scientists. An early pre‐funding application discussion with two patients, in person and on Skype, clarified a study patient advisory framework and also brought in one of those patients as a co‐applicant. Two years later a co‐produced suite of accessible information added to better public understanding of the uses of patient data through ESORT's ‘discovery’. ESORT has been a genuine partnership sharing power and decision making from the very first patient conversation. (P. C.)

## 3 DISCUSSION

This paper describes how plans and methods for PPI were modified during the pandemic. It considers this as an issue of general and ongoing importance for applied health research and does so within the context of studies evaluating emergency surgery for patients with common acute conditions. This context was specific but epitomises likely common complex challenges for PPI in an online environment. We draw from first‐hand accounts of patients, clinicians and non‐clinicians to consider our processes from different perspectives.

Here, we reflect on connections between our processes and two central concerns emerging in recent literature: representation and inclusion, and altered dynamics online.

### 3.1 Representation and inclusion

Representation and inclusion are strong conceptual threads in the online PPI literature. They are central to the value and fairness of patient and public consultations. While online approaches can open participation to some who were previously underrepresented, it is likely that for others they inhibit participation.

A positive example of widening representation through online PPI is the lifting of geographical barriers and travel requirements in particular for some people with health needs and for those with caring responsibilities.^9^, ^19^, ^20^ B. D. and E. G. indicated that they would not have joined face‐to‐face meetings, and speak of travel barriers in direct relation to complex health needs. For those with chronic debilitating conditions, who need drugs, equipment and facilities and face uncertainty about leaving home, avoiding travel lifts a major impediment to participation. This benefit extends to carers for people unable to travel: Molinari‐Ulate and colleagues^20^ found that carers of people living with dementia could participate in online sessions from home while also meeting caring responsibilities. Those with parenting responsibilities, as E. G. suggests, may find similar new opportunities to participate with online approaches.

It is important to consider the demands of the PPI sessions themselves, whether the online format might encourage or discourage participation, and shape the contributions that participants can make. Engler and colleagues^21^ asked participants to record views on a virtual whiteboard and reported that for their participants this ‘facilitated a feeling of participation and co‐production’, but for people with, for instance, visual impairments, low literacy or dyslexia, such aspects of online participation may be discomforting. Consultation methods need to be anchored to their context and should create a satisfying experience for participants. Two ESORT Study panellists had visual impairments but only one self‐identified and received our support. We did not consider making available a translator, signer or information in a dedicated format such as Braille or an Easy Read document. We should have been proactive in offering support to prepare and participate in the meetings and reduce the risk of biases and exclusion.

A major challenge within our PPI sessions was to facilitate discussion of unfamiliar topics including the use of routine health data and related outcome measures. We found that providing materials designed to engage, to meet different learning styles, and with embedded accessibility functions, helped the panel tackle complicated subjects. The materials were central to our approach but undoubtedly demanded time and effort from the panellists. It is likely that they would be challenging for those uncomfortable with reading, or those who are unfamiliar with digital documents.

Online approaches may exacerbate health and social inequalities known to already affect representation in PPI.^5^ Examples of potential risk factors for exclusion include language barriers,^19^ and cognitive ability for people with conditions such as dementia.^20^ Online PPI's dependence on material goods and skills creates particular concerns that digital poverty and low digital literacy, themselves markers of inequalities, will drive further exclusion,^8^, ^19^ marginalisation^22^, ^23^ and disenfranchisement.^24^ In response, PPI partners have been provided with equipment and funds to purchase data to facilitate involvement^21^ and offered digital skills training.^9^ Hybrid approaches might allow different participation modes.^20^ The digital divide is however a significant threat to the validity of online PPI and methodological research is needed to address the challenges involved.

### 3.2 Altered dynamics

Evidence of the impact of the altered dynamics of the online environment is mixed, with benefits for some^9^ and limitations for others.^5^ We found what one panellist referred to as a ‘democratising’ effect. Instead of a meeting room with a chairperson and audience, with speakers presenting slides from positions of authority, all participants in our meetings appeared onscreen equally in random order. Most joined from home. There were no suits or formal clothes. Everyone was listed by the first name without titles. All took turns to speak, emphasised by hand raising. There was space for panellists to ask the ESORT team questions and vice versa. Others have similarly observed a reduction in ‘power asymmetries between “experts” and the “public”’^19^ and disruption of ‘the hierarchy of speakers and disproportionately dominant contributors’.^9^

Some of this relates to platform functionality, in particular, hand‐raising and turn‐taking for all,^9^ and the ability to use chat functions to comment. Jones and colleagues^19^ argue that these features gave participants ‘confidence that their contribution will be acknowledged in an appropriate manner’. There is some dissent, however: difficulties in reading body language and picking up prompts^5^ have been cited as challenges to the discussion, and one study found that where the turn‐taking was not observed, this was experienced negatively.^19^

One potential challenge is that the online format may raise new obstacles to interaction given reduced opportunities for informal encounters that can support group formation. We aimed to address this with virtual coffee and smaller groups; others have gone further towards building ‘personal relationships’ through telephone contact, technical support and setting up the atmosphere of a break by sending drinks and snacks to participants' homes for use on the day.^21^

PPI processes and the online environment should be safe and comfortable for all. Some topics will invite participation by those with significant health needs or vulnerabilities and/or their carers. Participants have indicated a preference for meeting environments in which they are not required to maintain a constant visible presence.^24^ This offers control over what is and is not shared. Cameras and microphones can be discreetly turned off to deal with distress, discomfort, pain, equipment or to give or receive carer support. Parents may step away as needed when joining from home. This ability to self‐manage is especially important in sensitive situations, as addressing emotional needs in online settings is a possible challenge^25^; it may be difficult to see someone struggling emotionally and to reach out and offer support. This does not though remove the responsibility to respond to distress triggered by PPI discussions. Breakout rooms could be offered to allow participants to talk to a designated support person. Meeting set‐up materials might walk participants through these options. These actions do however require proficiency in online environments.

The facilitator can influence the quality of proceedings and discussion^20^ by being welcoming, observant and responsive. They may be an active presence or may miss hands raised or important comments in chat boxes. Multiple facilitators per meeting have been recommended, and facilitator training is proposed as essential to the quality of online PPI.^19^ We suggest that developing and attending such training would mark a commitment to making the altered dynamic of the online PPI environment a safe and effective space.

## 4 CONCLUSIONS

PPI is evolving and new online approaches may help avoid some of the biases within traditional PPI.^26^ Chew‐Graham argues that ‘we need to continue to do things differently’.^27^ For the ESORT Studies, an online approach encouraged participation by some who would otherwise have been excluded. It generated broad discussion and helped refine research outputs. There may be important methodological challenges in studies where topics are sensitive, or PPI partners are marginalised or vulnerable.

While our experience of developing online PPI methods raises some conceptual and practical issues likely to be of wider relevance, it must be recognised that the methodological choices for other studies will be context‐dependent. Research teams will need to consider the interplay of research questions, the PPI population, and online proficiency, to navigate their particular challenges. While we cannot speak for every panellist, as there may have been needs and dissatisfaction not shared with the team, we felt that, for these studies, in this context, with these methods and participants, online meetings facilitated and supported collaboration and contributions.

Online PPI is not simply a matter of moving people and materials to a different setting. For complex topics, there is a disconcerting trade‐off between developing methods to promote engagement, and placing additional demands that may act as a barrier to participation. It is important that wider methodological challenges are addressed, and researchers continue to provide detailed examples of novel and inclusive methods for discussion and empirical study.

## AUTHOR CONTRIBUTIONS

All listed authors were involved in the development or implementation of the methods described for one or more Patient and Public Involvement events carried out for the Emergency Surgery OR noT Studies. All contributed to the conceptualisation of the manuscript and have agreed to the final submitted version. Claire Snowdon, Elizabeth Silver and Richard Grieve led the preparation of the manuscript. Brian Devlin, Emma Greenwood, Susan Moug, Richard Grieve and Paul Charlton wrote accounts of their involvement for inclusion in the manuscript.

## CONFLICT OF INTEREST STATEMENT

The authors declare no conflict of interest.

## Data Availability

Data sharing is not applicable to this article as no datasets were generated or analysed during the current study.
